# Recent advances in mass spectrometry based clinical proteomics: applications to cancer research

**DOI:** 10.1186/s12014-020-09283-w

**Published:** 2020-05-24

**Authors:** Andrew Macklin, Shahbaz Khan, Thomas Kislinger

**Affiliations:** 1 Princess Margaret Cancer Centre, University Health Network, Toronto, Canada; 2 Department of Medical Biophysics, University of Toronto, Toronto, Canada

**Keywords:** Clinical proteomics, Mass spectrometry, Cancer, Biomarker discovery, Targeted assay, Proteogenomics

## Abstract

Cancer biomarkers have transformed current practices in the oncology clinic. Continued discovery and validation are crucial for improving early diagnosis, risk stratification, and monitoring patient response to treatment. Profiling of the tumour genome and transcriptome are now established tools for the discovery of novel biomarkers, but alterations in proteome expression are more likely to reflect changes in tumour pathophysiology. In the past, clinical diagnostics have strongly relied on antibody-based detection strategies, but these methods carry certain limitations. Mass spectrometry (MS) is a powerful method that enables increasingly comprehensive insights into changes of the proteome to advance personalized medicine. In this review, recent improvements in MS-based clinical proteomics are highlighted with a focus on oncology. We will provide a detailed overview of clinically relevant samples types, as well as, consideration for sample preparation methods, protein quantitation strategies, MS configurations, and data analysis pipelines currently available to researchers. Critical consideration of each step is necessary to address the pressing clinical questions that advance cancer patient diagnosis and prognosis. While the majority of studies focus on the discovery of clinically-relevant biomarkers, there is a growing demand for rigorous biomarker validation. These studies focus on high-throughput targeted MS assays and multi-centre studies with standardized protocols. Additionally, improvements in MS sensitivity are opening the door to new classes of tumour-specific proteoforms including post-translational modifications and variants originating from genomic aberrations. Overlaying proteomic data to complement genomic and transcriptomic datasets forges the growing field of proteogenomics, which shows great potential to improve our understanding of cancer biology. Overall, these advancements not only solidify MS-based clinical proteomics’ integral position in cancer research, but also accelerate the shift towards becoming a regular component of routine analysis and clinical practice.

## Background

Cancer is the second leading cause of death and poses a major problem to healthcare systems worldwide. The prevalence of cancer remains stable with an estimated 1.7 million new cases, resulting in 600,000 new deaths, in 2018 in the United States alone [1]. Currently, clinical practices are being improved by research on early detection methods, appropriate classification of risk groups and treatment efficacies. Much of this research has characterized tumours at the molecular level using a systems biology approach aimed at biomarker discovery. The National Cancer Institute (NCI) defines a biomarker as a biological molecule found in blood, other body fluids, or tissues that provides an indication of a normal or abnormal process, or of a condition or of a disease. They are used in the early detection, diagnosis, prognosis and treatment selection in the oncology clinic. The routine measurement of biomarkers and better treatment options in oncology clinics have led to a gradual reduction in cancer mortality rates with an estimated 1.5% annual decline, amounting to a 26% decrease over the past three decades [1].

Other fields of clinical research attempt to elucidate molecular differences between cancer cases and healthy controls or different stages of cancers as the disease progresses. These include genomics and transcriptomics that have identified numerous cancer-driving genes. While these omics datasets have demonstrated the ability to compare and contrast different clinical cancer groups, one limitation is that these changes do not necessarily directly translate to our understanding of disease biology. On the other hand, proteins are the biomolecules that directly carry out most biological processes suggesting they are ideal predictors of disease progression [2]. Additionally, proteins are the active targets of most cancer therapeutics including the growing field of immunotherapies. This makes clinical proteomics a growing field in molecular clinical research: the large-scale study of proteins, including their expression, functions and structure, and applying the findings to improve patient care.

Multiple studies have shown that globally mRNA expression is positively, but weakly, correlated with protein expression [3–6]. This may be one reason why results from transcriptomic studies have translated to the clinic with mixed results and support the implementation of additional (and complementary) research in clinical proteomics. This discordance arises from the highly dynamic and complex nature of proteome regulation. Protein expression is affected by alternative splicing, SNP’s (which translate to different proteoforms) and transcript degradation, as well as protein-level processes such as protein–protein interactions, degradation rates and post-translational modifications (PTMs) [7, 8]. Accurate protein detection techniques are required for routine clinical analysis.

There currently exists a strong bias towards antibody-based techniques for the detection of clinically-relevant proteins. ELISA is commonly used to quantify protein biomarkers in a variety of biofluids, with ongoing improvements, such as Prostate-specific antigen (PSA) in the blood of suspected prostate cancer (PCa) patients as low as one hundred picograms per millilitre [9]. Immunohistochemistry (IHC) stains tissues to provide spatial information regarding well-established cancer markers. For example, the protein markers HER2, ER and PgR are used to classify breast cancer subtypes which has significant implications in selecting an appropriate treatment. ER and PgR-positive tumors are treated by endocrine therapy while HER2-positive status is a prerequisite for targeted immunotherapy [10]. IHC is also useful in discerning tissue physiologies associated with poor prognosis or treatment response. These include tissue hypoxia using markers such as HIF1α [11], or staining for infiltrating immune cells such CD69+ activated lymphocytes in melanoma [12]. Fluorescence activated cell sorting (FACS) uses antibodies to detect a small panel of protein markers and determine heterogeneity amongst a population of cells. The main advantage of antibodies is the detection specificity they provide but their application comes with several disadvantages: cost of development, availability, quality, and ability to be multiplexed. The need for higher-throughput techniques that capture a wider swath of the cancer proteomic landscape have opened the door for mass spectrometry (MS)-based techniques in the oncology clinic. Rapid technological advancements on multiple fronts including sample preparation, peptide separation, MS-detection, and data analysis have all been essential for the robust quantitation of proteins from complex clinical samples.

In this review, we highlight relevant literature related to MS-based clinical proteomics with a specific focus on cancer research. We specifically focus on clinical sample types, sample preparation techniques, MS configurations and protein quantitation strategies. To limit this review to a more manageable scope we further describe notable studies that specifically investigate the proteomes of cancer tissues and bodily fluids. While we attempted to be as complete and inclusive as possible, we apologize to authors whose papers were not cited as part of this review.

## Recent advances in clinical proteomic methodologies

### Clinical sample preparation methods

A wide array of sample types has been analyzed by clinical proteomics. First and foremost, larger cohorts of primary patient materials in the form of tissue samples are becoming increasingly feasible, due to improvements in biobanking and proteomics technologies. As a result, the direct proteomic investigation of clinical tissues is becoming increasingly popular. Preservation of the tissue’s proteome dynamics is critical from time of surgical resection to the protein digestion stage, and there are a few methods of doing so: fresh frozen (FF), formalin-fixed paraffin embedded (FFPE), and optimal cutting temperature embedded (OCT). One important caveat for consideration in clinical tissue-based proteomics is that surgical procedures could possibly take hours from the time a patient is admitted to the operating room to the point of sample retrieval and preservation. How this affects a tissue proteome is currently poorly understood. More rapid procedures such as needle biopsies have the potential to overcome some of these complications but provide significantly lower amounts of tissue for proteome analysis. While FF is the preservation method of choice from a proteome coverage perspective, FFPE tissues have been banked for decades, providing extensive clinical follow-up and an invaluable resource for clinical proteomics. Previously, cross-linking-based modifications produced insufficient proteomic coverage for global proteomic studies of FFPE tissues. Improvements in sample preparation have led to more efficient de-crosslinking of fixed proteins, and as a result, greater protein availability for digestion. Tissue samples can be further prepared by laser-capture microdissection (LCM) to add an element of spatial resolution. This allows different regions of the same tissue sample to be compared, whether normal adjacent or tumorous.

While tissue samples have the potential to provide novel biological insights, many clinical proteomics studies have aimed to discover novel biomarkers, ideally in clinical samples that are obtainable in a non-invasive or minimally invasive manner (i.e. liquid biopsies). The most commonly analyzed biofluids are blood (plasma, serum) and urine, but other biofluids that have been analyzed by proteomics include post-digital rectal examination urine [13], expressed prostatic secretions [14], saliva [15], tears [16], cerebrospinal fluid (CSF) [17], and ascites [18, 19] to name a few. The promise of analyzing patients’ body fluids is that disease-relevant changes in molecular markers such as cfDNA, RNA, proteins, lipids and metabolites are reflected in the fluid sample. Therefore, a liquid biopsy sample can be collected in a renewable manner for longitudinal studies that monitor cancer progression and a patient’s response to treatment.

**Fig. 1:**
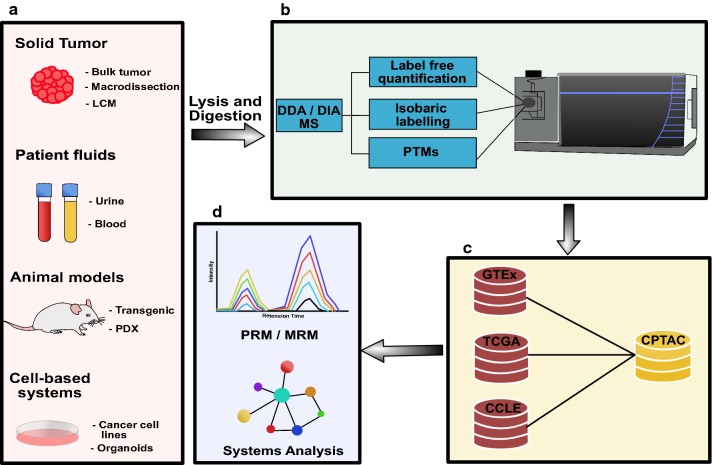
Overview of clinical cancer proteomics strategies. **a** Various sample types are used for clinical proteomics. These include solid tumor tissues, patient body fluids, animal models and cell-based systems. Tumor tissues are obtained either as surgically resected samples or are biopsy based. There are a number of tissue processing approaches available, which include the analysis of “bulk” tissue or preferentially after pathological inspection, tissue macro-dissection or laser capture microdissection (LCM). Patient fluids are a popular source for the discovery of biomarkers. The most commonly used patient body fluids include blood (processed to plasma or serum) and urine. Animal models are a popular in vivo model system for clinical proteomics. The most common models include transgenic disease models and patient-derived xenografts (PDX). Cell-based systems continue to be popular model systems in cancer biology. They include immortalized cancer cell lines or more sophisticated organoid systems that are established using defined culture conditions and primary patient material. Samples obtain from these sources are homogenized and proteolytically digested prior to proteomic analyses (i.e. bottom-up proteomics). **b** Proteomic analyses can use several well-established workflows. These include label-free proteomics (LFQ), isobaric labelling strategies or the specific enrichment of post-translational modification such as phosphorylation, ubiquitination, glycosylation, etc. **c** Integration of proteomics data with publicly available resources such as the CPTAC proteomics data or transcriptional profiles from GTEx, CCLE and TCGA can be used for biomarker prioritization. **d** Bioinformatics analyses (clustering, enrichment, pathways, etc.) are used to extract biological content or further prioritize candidates for targeted proteomics validation, using multiple reaction monitoring (MRM) and Parallel reaction monitoring (PRM)

Clinical sample cohorts of the past were often underpowered due to biobanking limitations (i.e. availability of high-quality, richly annotated samples). As a result, various model systems have been developed to facilitate the discovery of new biomarkers or aid in characterizing proteins of interest, as in Fig. 1. These models include transgenic animal models, immortalized cancer cell lines, primary cell lines and xenograft models. Cell lines of various cancer subtypes can be grown in 2D on cell culture dishes, either directly on plastic or on various matrices (i.e. collagen), or under more sophisticated 3D conditions (i.e. embedded in Matrigel). More recently, primary cells have been established as organoid models that more accurately mimic three-dimensional tumour development [13]. Alternatively, cancer cell lines or patient-derived tumors can also be engrafted into immunocompromised mice to generate so-called cell line-derived xenografts (CDX) or patient-derived xenografts (PDX), respectively [20–22]. These xenograft models are thought to more accurately recapitulate human tumor specimens, due to the presence of matrix components and stromal cells (i.e. vascular cells, fibroblasts, etc.), but still lack important contributions from the immune system. Model systems play a critical role in allowing for an expansion of clinical proteomic studies to gain more functional insights. As such, data from representative model systems can act as a complement to data obtained from clinical samples.

Sample preparation plays an important role in the proteomic characterization of clinical samples and rigorous standard operating procedures need to be established in order to get relevant information on the complex biological processes that lead to cancer progression. There is no universal protocol for proteomic sample preparation, but rather the selected strategy should be optimized/selected based on the proteomic complexity, the available quantity of sample and the goal of the study. The first step in sample preparation for MS includes lysis and extraction of proteins from the clinical samples. This includes extraction reagents such as different organic solvents and detergents followed by tissue disruption techniques such as freeze–thaw cycles, sonication or mechanical disruption to maximise the protein extraction and solubilisation. Organic solvents solubilise and denature proteins and can be easily removed by evaporation using lyophilization. TFE (2,2,2-Trifluoroethanol) based lysis and extraction on nano-scale (30 µg) and macro-scale (> 100 µg) input materials gave comparable protein detection rates relative to the traditional detergent-based methods [23]. While the use of other organic solvents such as acetonitrile have been reported, TFE-based sample preparation in clinical studies have been shown in the study of ovarian cancer [24] and PCa tissues [6].

Denaturants (urea and guanidine HCl), ionic detergents (SDS, SDC), and non-ionic detergents (Triton X-100, NP-40) act to efficiently lyse cells and solubilise protein complexes, especially membrane proteins. The disadvantage of detergent use is their difficult removal from samples for downstream MS applications that could lead to peptide ion suppression. Detergents also tend to deposit in the electrospray emitters and liquid chromatography lines, C18 chromatography columns, and in the MS instrument front-end causing added maintenance. Many MS-compatible, commercially available detergents have been reported and widely used including Rapigest (Waters), ProteaseMax (Promega), Invitrosol (Thermo). These detergents degrade with the addition of heat or acidic pH conditions; hence reducing problems described above. Recently different protein purification techniques have been developed with increased efficiency, reduce protein digestion time requirements and minimized sample losses as described below.

### FASP, MStern and S-trap

The anionic surfactant sodium dodecyl sulfate (SDS) is an excellent agent to solubilise proteins but possess limited compatibility with MS applications. The removal of SDS from the peptide sample has proven to be a major barrier with conventional methods. Recently developed sample preparation methods focus on using SDS and other denaturants as the solubilization agent and their removal through various membrane-based protein capture techniques. One of these developed methods is filter aided sample preparation (FASP) first described by Manza et al. [25] and further characterized by Wisniewski et al. [26]. FASP uses molecular weight (MW) filtration to bind proteins to a nitrocellulose filter, while lower MW analytes pass through the filter. Consecutive urea washes facilitate SDS removal, followed by on-filter digestion and peptide elution. This technique reduces sample preparation time and sample loss while maintaining the advantages of using SDS for improved proteome coverage. One of FASP’s limitations is a reduced binding efficiency with small quantities of starting material with greater sample losses [27]. Additionally, the small membrane pore size in FASP requires higher spinning speeds which makes it time consuming in the 96-well format. Distler et al. [28] made minor modifications to the FASP protocol to minimize potential losses of input material and reduce processing time. Alkylation and reduction were performed on-filter and further washes with MS-compatible volatile salts such as ammonium bicarbonate leading to highly purified samples. FASP has been utilised in different clinical tissue proteomics studies including colorectal cancer (CRC) FFPE samples [29, 30], PCa FFPE tissue samples [31] and FFPE samples of PCa bone metastases [32], to name a few.

Clinical proteomics studies consist of increasingly large cohorts which require efficient and timely sample preparation; which has been facilitated by the development of 96-plate format techniques. The MStern method was developed to overcome the problem of slow liquid transfer through nitrocellulose membrane. MStern uses hydrophobic PVDF membranes with significantly larger pore sizes allowing for improved liquid transfer and more efficient protein adsorption relative to nitrocellulose [33]. A vacuum system is used for passing the samples through the membrane more effectively than centrifugation. Similarly to FASP, MStern involves reduction, alkylation and digestion on the same membrane. Apart from the speed and efficiency of MStern, peptides are not eluted with a high salt concentration. Rather, they are eluted by acetonitrile and formic acid which limits the need for extra desalting steps. The one limitation of MStern is that the binding capacity of each well is 25 µg compared to < 400 μg for FASP. A study by Berger et al. compared MStern and FASP techniques using urine, CSF and whole cell lysate samples with varying amounts of starting material. MStern showed a comparable number of protein detections while saving 9.5 h of processing time [33].

Another recently developed membrane-based method that uses a similar principle as FASP is called suspension trapping (S-trap) [34]. S-Trap packed filters consist of quartz fibers packed with a larger pore size compared to FASP. The other protocol modifications include the use of higher SDS concentrations (5%) in the lysis method. The addition of methanol and phosphoric acid causes the formation of protein particulates which are trapped by the filter. Similarly, to FASP and MStern, reduction, alkylation and digestion are done directly on the filters. The comparative study to evaluate the overall efficiency of S-Trap, MStern and FASP showed that S-Trap and FASP provided the greatest number of protein detections compared to the polyvinylidene difluoride (PVDF) method. The digestion efficiency was greatest for S-trap which reported the lowest number of missed cleavages [35]. Another study showed that S-Trap outperformed FASP in terms of protein detection due to a higher digestion efficiency [36].

### SP3 and iST

Clinical tissue samples are sometimes challenging to process due to their small size, particularly LCM samples. These samples require efficient sample processing techniques to ensure limited sample losses and maximal extraction of the proteins to maximize proteome coverage. The solid-phase-enhanced sample preparation (SP3) method was developed with these limitations in mind. Originally described by Hughes et al. [37] the method uses paramagnetic beads which are coated with hydrophilic carboxylate groups. The beads are compatible with various detergents and organic solvents including SDS, urea, TFE and acetonitrile (ACN) [27, 38]. The proteins are immobilized to the charged carboxylate groups in the presence of an organic solvent with acidic or basic pH. After the immobilisation of the proteins, detergents are removed with high organic content washes, followed by on-bead digestion. Eluted peptides can be directly introduced into the MS without the need for desalting. SP3 protocol has been further modified to improve the efficiency and its reproducibility. These studies showed that binding efficiency of proteins are lower in acidic conditions as compared to neutral pH [39]. Furthermore, using ethanol in neutral pH during protein binding resulted in better protein recovery compared to ACN in acidic pH [40]. The strength of SP3 beads lie in providing a platform for efficient protein binding from minute amounts of starting material; with all sample preparation steps happening in one tube to limit potential losses. This method has been scaled to an 96-well automated robot liquid handling platform for robust reproducibility and through-put [41]. Several clinical studies using patient derived samples have used the SP3 method, including ovarian cancer [42], CRC [43].

The in-StageTip (iST) method developed by Kulak et al. [44] in 2014 is another sample preparation workflow, which is compatible with low input material. The method focuses on using a single stage tip enclosed by a barrier to perform multiple sample processing steps to minimize sample losses and to provide better proteome coverage. In the iST workflow, a pipette tip is inserted with a reversed-phase membrane barrier at the bottom. The sample in the tip is introduced from the top, where they can be lysed through heating or sonication. The sample is then denatured, alkylated and digested. The membrane at the bottom of the tip is then used for peptide clean-up. Alternatively, samples can also be fractionated on the same tip. The iST workflow shows high performance handling ultra-low amount of material, but due to the reverse-phase membrane barrier the iST is incompatible to use detergents (SDS) and organic solvents (TFE) for lysis [37].

The work by Sielaff et al. [39] showed an independent comparison between commercially available iST, FASP and SP3 sample preparation strategies using minute amounts of starting material. Initially, varying amounts of HELA lysates (1–20 µg) were used to process these samples. The results showed that all three methods performed similarly with respect to the numbers of protein detections and reproducibility. Reduction of the input < 10 µg resulted in SP3 and iST providing similar proteome coverage, whereas FASP showed a decrease in performance. Furthermore, clinically relevant FACS immune cells (25,000) were processed in triplicates using these three methods. The highest number of proteins were detected through SP3 method giving an average detection of 3152, followed by 2343 proteins by iST and FASP detecting 109 proteins. These results suggest that SP3 and iST methods are suitable for low input starting material while FASP may not be feasible for ultra-low amounts of starting material.

### Front-end MS developments

The field of clinical proteomics has seen advancements at the sample preparation stages, as well as in MS technology. MS-based proteomics’ emergence can be attributed to the development of soft ionization techniques such as electrospray ionization (ESI) and matrix-assisted laser desorption ionization (MALDI). Further advancements in front end technologies have propelled clinical proteomics to further depths of the human proteome. ESI continues to rely on well-established reversed-phase nano-LC technologies, or combined with capillary electrophoresis [45], and is hence more practical for discovery-based experiments. Orthogonal peptide separation techniques have grown in popularity amongst clinical proteomics research applications. For increased proteome coverage over a single-shot experiment, a peptide pool can be fractionated by basic reverse-phase LC or strong cation exchange chromatography. For example, peptides from lung cancer cell lines resistant and sensitive to tyrosine-inhibitor treatment were fractionated and produced 39% more detections per protein than a single shot analysis [46]. The increased burden of LC–MS time requirements can be reduced by strategic concatenation of non-sequential fractions. Additionally, this strategy comes with increased sample handling and loss of peptides; an important consideration when clinical samples may be limited in protein input and availability [46]. In the gas phase, peptides can be further separated by ion mobility to achieve greater proteome coverage. These technologies include high-field asymmetric ion mobility spectrometry (FAIMS) and trapped ion mobility spectrometry (TIMS) aim to reduce MS1 complexity and thus MS2 contamination from co-eluted and co-isolated peptides [47, 48]. These technologies have demonstrated a 30% increase in peptide detections from routine analysis of cancer cell lines [49] and this improvement translates similarly to clinically-relevant specimens. Overall, the improvement of front-end peptide separation techniques is ongoing to meet the goal of increased proteome coverage.

### MS scanning modes

The majority of discovery-based clinical proteomic studies continue to depend on data-dependent acquisition (DDA) to identify potential biomarkers or gain biological insights. This approach has some benefits including well established instrument operation, data analysis and processing pipelines. Additionally, DDA includes the option of label-dependent quantitation and associated multiplexing. On the downside, DDA is hampered by low inter-sample reproducibility of peptide detection due to random sampling, thus creating a “missing value” problem. While the premise of DDA has remained largely unchanged, improvements in the instrument’s efficiency have stemmed from the development of the BoxCar data-acquisition method [50]. Unlike past DDA advancements that focus on the MS2-level, BoxCar sequentially fills narrow m/z windows to increase the ion injection time more than ten-fold. The increase in ion collection significantly increases the signal-to-noise and overcomes issues of abundant peptides dominating the MS1 spectrum, while less abundant, co-eluting peptides were less likely to be selected for MS2. As a result, 90% of a cancer cell line proteome was detected in a 1-h analysis as opposed to 24 fractions. Reproducibility was also high, as the majority of proteins were quantified in all ten replicates. BoxCar will undoubtedly be applied to cancer-relevant studies in the near-future [51]. Another recent advancement in DDA is the MaxQuant.Live software that combines aspects of global and targeted MS. It applies on-the-fly mass, retention time and intensity calibration and controls the Orbitrap mass analyzer to predict the detection of significantly more precursors in real-time [52].

Due to the shortcomings of DDA, the field of clinical proteomics is observing a shift towards data-independent acquisition (DIA), a method that was originally described by Purvine et al. [53] and further reported by Venable et al. [54]. In DDA, the N most intense peptide precursors in a survey MS1 scan are selected for sequential fragmentation and MS2 detection. Whereas in Sequential Window Acquisition of All Theoretical Mass Spectra (SWATH-MS), a variation of classic DIA, all theoretical peptides in a sample are sequentially fragmented in narrow m/z windows to yield more complex MS2 spectra [55, 56]. These spectra are then matched to a pre-defined, empirically determined, spectral library of peptides with the goal of the library having achieved maximum proteome depth through extensive peptide fractionation. Additionally, peptide sequencing and quantitation are more robust since stochastic peptide detections as in DDA are no longer a concern. Current data analysis platforms such as Spectronaut Pulsar [57], OpenSWATH [58], and Scaffold EncyclopeDIA [59] (among others) are sufficient in de-convoluting the MS2 spectra to peptide sequences, at a rate comparable to DDA. Further improvements will allow less abundant peptides to be confidently matched. Clinical samples with greater proteomic diversity, such as tissues, make this MS2 complexity issue more troublesome. On the other hand, clinical samples of less complexity are well-suited for DIA analysis, such as urine. DIA’s reproducibility was tested across 11 laboratories using a benchmarked sample of stable heavy isotope labelled peptides spiked into a cell lysate digest with 91% of proteins detected across all centers [60]. Furthermore, public databases such as SWATHAtlas will continue to increase spectral library availability and increase DIA’s ascension in clinical proteomics [61, 62].

### Fragmentation and MS detection techniques

MS instruments continue to show versatility with various peptide fragmentation and detection configurations. Collision-induced dissociation (CID) was first introduced in 1981 and is one the most fundamental fragmentation techniques in proteomics [63]. The ionized peptides are passed through a vacuum chamber where they collide with a neutral gas such as nitrogen, helium or argon. The vibrational energy cleaves the C–N (peptide bonds) to generate b and y ions series, followed by mass analyzer detection. Higher-energy collision dissociation (HCD) which is essentially a vendor-specific term for CID is often used in Orbitraps and hybrid LTQ-Orbitrap mass spectrometers which combine the cycle speed and sensitivity of the linear ion trap with the mass accuracy and resolution of the Orbitrap. Precursor ions are shuttled from the C-trap to the collision cell where the ions are similarly fragmented by a neutral gas [64]. The fragments are then transmitted to the Orbitrap analyzer resulting in improved MS2 spectrum quality, particularly with lower molecular mass [65].

Electron-transfer dissociation (ETD) is an ion–ion collision fragmentation-based method where cations (peptides or proteins) collide with charged radical reagent anions [66]. ETD is particularly useful in the study of diverse modifications because PTM integrity is preserved while still achieving the backbone fragmentation necessary for peptide detection. Hybrid ETD fragmentation methods like ETD-HCD have been reported where precursors sequentially undergo both types of fragmentation to yield b/y and c/z type fragment ions in a single spectrum [67]. Additionally, ETD is suitable for the study of top-down proteomics due their high-cationic nature including intact proteins, their PTMs, and protein–protein interactions. One such application is the study of engineered antibodies which have shown potential to be used as cancer therapies [68]. ETD-based MS methods have been reported in the literature to characterize monoclonal antibodies (mAb) and antibody drug conjugates (ADC) [69–74].

### Protein quantitation

There is a large number of global and targeted protein quantitation approaches available, each with their unique sets of advantages and disadvantages. These can be categorized as relative or absolute quantitation, of which the former can be further divided into label-dependent and label-free techniques.

Relative quantitation often depends on the use of stable-isotopic labels that result in covalently derivatized peptides. Past methods include metabolic and chemical labelling, such as dimethylation and Stable Isotope Labeling with Amino Acids in Cell Culture (SILAC) which rely on MS1-level quantitation, but they are most applicable to cell line-based studies [75, 76]. Additionally, SILAC-based methods have also been adopted to in vivo studies, termed Super-SILAC, where heavy labelled cell line lysates are spiked into tumor tissue lysates as a global control [77]. This approach was used for histone PTM quantitation in breast cancer FFPE samples [78].

More recent labelling strategies, such as Tandem mass tag (TMT) and isobaric tag for relative and absolute quantitation (iTRAQ), have gained popularity as these techniques apply isobaric tags providing MS2-level relative quantitation while boosting the MS1 signal intensity to improve chances of peptide detection [79, 80]. A potential problem with these approaches is the co-isolation of precursor peptides that results in ratio compression. Improvements in instrument resolution with narrower isolation widths, and the combination of isobaric tags with ion mobility to reduce co-isolation have significantly improved the quality of these experiments. One notable advantage of using TMT in a clinical proteomic setting is the high degree of multiplexing, up to 16-plex, to significantly reduce the LC–MS time requirements of analyzing increasingly large patient cohorts. Potential caveats of TMT -based approaches are that extensive peptide-based fractionation is required to obtain deep proteome profiles and that 1–2 TMT channels are usually dedicated to a global control (i.e. a combined lysate of all clinical lysates analyzed). This reduces the ability of individual projects to be effectively compared against each other. More recently, the ratio compression of TMT labels and thus limited dynamic range of quantitative proteomic data can be overcome with further fragmentation. MS3-level quantitation further removes co-isolating and co-eluting peptides with the use of SPS (synchronous precursor selection) with only a marginal decrease in ion signal intensity [81].

The use of stable-isotopic labels does create added expenses in clinical proteomic studies. As an alternative, recent advances in data computation have given rise to powerful label-free proteomic techniques, with sample peptides often separated over long LC gradients. Relative protein abundances are derived from MS1-level peptide peak integration using software like MaxQuant [82], Proteome Discoverer and Skyline [83]. This allows for wider dynamic ranges spanning several orders of magnitude that are difficult to achieve with label-based techniques, albeit at a loss of precision (i.e. reproducibility). Label-free quantitation is more applicable to clinical proteomics due to the inter-patient and intra-patient variability of protein expression. Label-free strategies are applied to both DDA and DIA scanning modes. Latosinka et al. [84] compared label-free and isobaric tag (iTRAQ) strategies in muscle-invasive and non-muscle-invasive bladder cancer tissues. It was concluded that both methods provide comparable proteome coverages and proportional quantitative data, but label-free DDA identified a greater number of differentially expressed proteins.

In general, relative quantitation strategies are used for discovery-based clinical proteomics. Once proteins of interest have been identified they require further validation. While antibody-based techniques such as ELISA fulfill this role, MS-based targeted assays are well suited for validation, especially if no suitable antibodies are available. The most commonly used method is called multiple-reaction monitoring mass spectrometry (MRM-MS). This assay is well established for small molecules and peptides and is carried out using triple quadrupole mass analyzers. MRM-MS assays require prior knowledge of the parent ion (MS1) and fragment ion (MS/MS) mass-to-charge ratios. The combination of a parent ion and 3–5 associated fragment ions, termed transitions, are selected by the quadrupole. Quantitative data is obtained by measuring the area under the curve of the transitions extracted ion chromatograms. More recently, targeted assays have also been developed using quadrupole Orbitrap mass analyzers—termed parallel reaction monitoring mass spectrometry (PRM-MS), which leverage the high-resolution, accurate-mass (HRAM) Orbitrap for increased specificity [85, 86]. Similar to MRM-MS assays, prior knowledge of parent ion mass-to-charge ratios is required to develop PRM-MS assays. Since all fragment ions are generated and recorded in PRM-MS assays, prior knowledge of individual transitions (fragment ion mass-to-charge ratios) is not required. Rather the best fragment ions can be selected from the full MS/MS spectrum afterwards. Extracted fragment ion chromatograms, similar to described above, are used for quantitation. If appropriate controls such as stable isotope labelled peptide standards are used, targeted proteomics assays can provide absolute quantitation. Importantly, each targeted assay must be carefully optimized to achieve optimal performance. This includes optimized chromatographic separation, collision energies to achieve maximal fragment ion signal-to-noise and dwell times (MRM) or ion fill times (PRM), as recently described [87]. Optimization of these parameters also depends on the total number of peptides targeted. Ideally, for each peptide the assay linearity, level of detection (LOD) and lowest level of quantification (LLOQ) are experimentally determined using synthetic peptides [88]. Furthermore, the mass spectrometer’s detection efforts can be further optimized by using retention time information to schedule MRM/PRM target detection. These strategies require robust chromatography schemes that can be monitored using standard index retention time (iRT) peptides This allows for a substantial increase in the number of targets an assay can accurately monitor. Advances in instrument detection speed now allow for hundreds of targets to be monitored in a single injection [87, 89]. As the door opens for routine monitoring of proteomic signatures in the oncology clinic, targeted PRM approaches show great promise due their ability to monitor progressively more targets in a dependable fashion.

## Tissue biopsy studies

### Discovery-based studies

In the context of clinical proteomics, tissue analysis provides the most accurate reflection of the tumour’s physiological state. As mentioned, recent advancements in LC–MS technology have continuously enabled increased proteome coverage with reliable quantitation. These studies now enable routine proteomics analyses of patient tumor tissues for biomarker discovery, discovery of biological pathways and integrations with available genomics/transcriptomics profiles.

Tissue-based proteomic strategies have been applied to the study of many cancer types, including prostate [90, 91], breast [77, 92, 93], melanoma [94, 95], lung [96–98], ovarian [99, 100], and oropharyngeal carcinoma [101]. The studies above can be summarized by common themes. Clinical proteomic studies often compare cancerous tissue samples with “healthy” adjacent controls from the same patient for potential diagnostic biomarkers. Meanwhile, comparisons between patients with varying stages of cancer are compared for prognostic information. Once a smaller number of candidate proteins have been identified, pathway analyses give insight into how these proteins are associated with tumorigenesis, proliferation, metastasis and other cancer-driving processes. This is often followed by antibody-based techniques that complement and validate the differential expression findings in a larger independent cohort. The studies detailed below follow these guidelines and are summarized in Table 1.

**Table 1:** Summary of select tissue-centric proteomic studies highlighted in this review

Protein quantitation	Tissue type	Sample preparation	MS Model	Clinical question	Proteins detected	Patient cohort	References
Label-free DDA	FFPE	FASP, SAX	QE	CRC, healthy tissue and adenoma	10,000	32	[26]
			QE	Malignant vs. benign prostate tissue	9000	36	[31]
			QE	Breast cancer heterogeneity and triple-negative subtypes	10,819	131	[92]
		FASP, SAX, Super-SILAC normalization	QE	ER-positive luminal breast cancer progression and metastasis	10,000	88	[77]
			QE	Breast cancer subtypes	10,000	40	[93]
			QE HF	Melanoma response to immunotherapy	10,300	116	[95]
		SDS	QE	Ovarian cancer chemosensivity and chemoresistance mediators	9000	25	[105]
		Phospho-enrichment	QE	Triple-negative breast cancer treatment outcomes	2643	34	[110]
		TFE	QE HF	Metabolic regulators of CAF’s in high-grade serous carcinoma	6944	107	[24]
		FASP, SAX, LCM	LTQ XL	Colon cancer with healthy matched	6000	6	[29]
		MudPIT	LTQ XL	HPV-positive and HPV negative oropharyngeal carcinomas	2633	53	[101]
	FF	FASP, SAX	QE	PCa bone metastasis characterization	5067	22	[32]
		Urea, SDS, CHCl_3_/MeOH precipitation	QE	Ovarian carcinoma histotypes	6360	20	[100]
		iST	QE HF	Primary urachal carcinoma, metastases and healthy tissue	5543	1	[96]
Label-free SWATH	OCT	Pressure-cycling technology, urea	5600 TOF	Intratumoural heterogeneity of PCa	6873	60	[90]
	FF		5600 TOF	Renal cell carcinoma and healthy controls	4624	18	[103]
			5600 TOF	Hepatocellular carcinoma and healthy adjacent control	2579	38	[104]
		FASP	5600 TOF	Breast cancer classification	2842	96	[102]
Isobaric Labelling	FFPE	SP3, RPF, TMT	OF	High grade serous and clear cell ovarian carcinomas	8167	20	[42]
	FF	PDX, iTRAQ	OV, QE	Basal and luminal-B breast cancer subtypes	8126	2	[134]
		FASP, RPF, iTRAQ	OV	Non-muscle invasive and muscle invasive bladder cancer	900	8	[84]
		RPF, glyco-enrichment, iTRAQ	OV	Ovarian high-grade serous carcinoma and benign cases	4817	6	[99]
		Urea, glyco- enrichment, iTRAQ	QE	Squamous cell carcinoma vs. adenocarcinoma and healthy controls	8337	18	[106]
		PDX, phospho-enrichment, TMT	OF Lumos	Luminal and basal breast cancer subtypes	7700	4	[109]

A major application of discovery-based proteomics in cancer tissues is the development of risk stratification and cancer subtyping systems. This has been demonstrated by label-free strategies with several recent examples of DIA applications. Bouchal et al. [102] performed SWATH-MS on frozen breast cancer tumours. An extensive spectral library contained reference spectra for 28,233 proteotypic peptides and their modified variants, attributing to more than 4400 proteins. The discovery cohort consisted of 96 tissues belonging to five well-established clinical subtypes. Proteomic analysis resulted in the consistent quantitation of 2842 protein groups, the majority of which were involved in well-established breast cancer pathways. For the most part, proteomic analysis confirmed the conventional subtype classifications, but highlighted intra-subtype heterogeneity to further create a protein-based classification system. Namely, proteins INPP4B, CDK1 and ERBB2 were found to be linked to ER and HER2 status, patient outcome, and their expression was further validated in cell line models. A renal cell proteomic study was performed by Guo et al. [103] who analyzed tumor and healthy control tissue biopsies from nine different renal cell carcinoma patients (18 samples). The SWATH-MS approach resulted in the quantitation of more than 2000 proteins with high reproducibility. As above, several detected proteins were differentially expressed in the tumor region, with 296 proteins upregulated in tumor samples compared to controls. SWATH-MS was also used in a biomarker discovery study on hepatocellular carcinoma (HCC) performed by Zhu et al. [104]. In this study 38 biopsies from 19 HCC patients with punches from paired tumor and benign regions. The data was analysed by OpenSWATH using a pan-human SWATH assay library which led to the detection of 2570 proteins. Once again, several proteins were differentially expressed in tumor samples compared to benign tumors. In particular, a putative biomarker, MCM7 which is believed to be involved in liver cancer progression was further confirmed through IHC.

Aforementioned label-dependent protein quantitation techniques have also been applied to tissue proteomic for improved risk stratification. Iglesias-Gato et al. [31] performed a proteome-wide study of PCa progression with Super-SILAC quantitation. With an aim of finding prognostic biomarkers. Twenty-eight FFPE prostate tumor samples from with a range of Gleason scores 6–9, and eight adjacent non-malignant samples, were prepared for MS analysis. The peptides were fractionated, and the MS runs resulted in the quantitative detection of over 9000 proteins, with an elevated expression of CPT2, COPA and MSK1/2 in tumour tissues compared to non-malignant region. These elevated proteins were reported to play a role in the regulation of cell proliferation. Comparisons between Gleason groups highlighted pro-neuropeptide Y (Pro-NPY) to be associated with poor patient outcome in intermediate and high-risk patients (Gleason score ≥ 7), which was confirmed by IHC in an independent cohort.

Aside from improving risk stratification, and thus patient survival, clinical proteomics can be used to identify new targets for improved treatment efficiency. Recently, a label-free, global proteomics approach was used to investigate the proteomes of 25 FFPE ovarian cancer tissues, stratified by chemo-resistance. More than 9000 proteins were detected and the differential expression of CT45 was related to a positive chemotherapy response. Little was known about CT45’s molecular function, thus phosphoproteomic analysis of cell line models was pursued and suggested that CT45 increases the repair response to DNA damage. Additionally, CT45 produces immunogenic peptides that recruit cytotoxic T cells and promote tumour killing. Therefore, clinical proteomics was a powerful tool in identifying a target for future immunotherapies [105].

As demonstrated in the study above, tissue global proteomic studies can be further extended to the investigation of PTMs. Yang et al. [106] studied the global and glycoproteome of non-small cell lung carcinoma subtypes. In total 18 patient samples consisting of three squamous cell carcinoma (SqCC) tumor samples with matched benign tissues, six adenocarcinoma (ADC) samples with five matched benign samples and one normal healthy tissue. The digested samples were iTRAQ labelled and enriched for *N*-glycopeptides followed by reversed-phase LC fractionation prior to shotgun MS analysis. Different protein and glycoprotein signatures were found in ADC and SqCC samples, with pathways distinguishing between tumour types.

Protein kinases play an important role in signal transduction pathways, and the dysregulation of these pathways is an intriguing area of study in cancer [107]. Modern MS instruments and recent phosphopeptide enrichment methods have allowed for the large-scale detection and quantification of thousands of phosphorylation sites. Phosphoproteomic studies in clinical samples allow the identification of aberrantly activated kinases and their downstream substrates, which would otherwise be undetectable by traditional shotgun proteomics, serving as potential therapeutic targets. Many studies have shown important roles in understanding the molecular mechanism of cancers governed by phosphorylation-mediated pathways [108, 109]. Zagorac et al. [110] performed label-free quantitative analysis of the triple negative breast cancer (TNBC) phosphoproteome and compared relapsed and non-relapsed patients. In total 34 patient samples were lysed, digested and enriched for phosphopeptides. Label free single-shot runs resulted in the detection of more than 10,000 phosphosites, corresponding to 2643 phosphoproteins. The analysis showed 159 phosphosites to have increased phosphorylation status. Several different kinases were found to be hyperactivated in relapsed samples compared to the non-relapsed ones through pathway enrichment analysis. Six kinases, PNKP, CDK6, PRKFCE, c-Kit, P70S6K were considered to play a role in the relapse of cancer and showed potential as prognostic markers. Inhibitors against these six kinases were studied in TNBC PDX models and cell lines which showed antitumor activities suggesting potential therapeutic targets in TNBC.

### Targeted and multi-site validation

Tissue proteomic studies to date have focused on discovery-based studies that highlight countless potential markers of cancer. We need to move towards rigorous validation of these targets if the field is to evolve towards routine analysis in the oncology clinic. This bridge is being formed by targeted MS methods. PRM of tissue lysates, or immuno-affinity pulldowns of specific proteins from lysates, will become more mainstream [111]. For example, a panel of 54 proteins involved in tumour suppression, drug metabolism and chemoresistance were monitored in FFPE tissue lysates from 50 metastatic CRC patients. This identified differentially expressed proteins that can be used to help guide eligibility of patients for clinical trials [112]. In a breast cancer tissue study, PRM assays were developed for proteins implicated in treatment sensitivity such as HER2, EGFR and PTEN with sub-femtomolar limits of quantitation. This was applied to monitor treatment effects in cell lines, PDX models, and extended to 46 frozen tissue lysates. Interestingly, a subset of the clinically annotated HER-2 positive tissues showed only minimal levels of HER2 by PRM [113]. Targeted LC–MS provides improved levels of detection and quantitation by only focussing on a subset of the proteome, but on occasion, particularly from complex tissue samples, target concentrations are still below reproducible levels of quantitation. Immuno-affinity enrichment of targets is then required to overcome this obstacle. Such a strategy was applied to the measurement of osteopontin in FFPE breast cancer tissues. Two rabbit IgGs were used to isolate two peptide targets from the lysates of normal tissue and breast cancer tumours. It was reproducibly demonstrated that the tumours contained 30× more osteopontin than normal healthy tissues using the first peptide and supported by the second peptide showing a 28× increase [114]. It is important to note that a tissue’s preservation method has an impact on targeted MS detection. Sprung et al. [115] targeted 114 peptides from FFPE and frozen tissues and reported that while the reproducibility of measurements was consistent between sample types, MRM signal intensities were reduced by 34% in FFPE samples.

When using the same sample preparation methods, LC–MS configuration and data analysis pipelines, considerable inter-lab reproducibility can be achieved. TMT-10 was used to multiplex PDX tissues derived from two breast cancer subtypes and compared across three independent laboratories. Overall, 7700 human-originating proteins were distinguishable from the 3100 mouse-derived stromal proteins with a maximum deviation across laboratories of 7%. Additionally, the TMT ratio between the two subtypes produced a minimum R^2^ correlation of 0.88. Phosphoproteomic results, with an average of 37,000 phosphosites quantified per sample, were less reproducible. The maximum deviation in phosphoproteome coverage was 24% with a R^2^ correlation of 0.72 [109]. Similar studies to assess inter-lab reproducibility in cancer cell lysates have been performed with similar results. Thirty stable-isotope labeled peptides were spiked into 1 µg digest of cell lysate and detected by SWATH-MS across 11 laboratories worldwide over a 1-week period. The inter-lab median CV of the standard peptide intensities was 47.3% in comparison to the inter-day and intra-day median CV’s at each site of 8.9% and 5.5%, respectively. 4000 proteins were detected in more than 80% of LC–MS runs. Of these proteins, the inter-lab median CV of protein intensity across sites was 22% [60]. Another study compared independently generated datasets. For example, one CPTAC CRC proteomic dataset generated by 2D LC–MS was compared to the proteome of another 40 CRC tissues prepared by GeLC–MS in a separate laboratory. The proteomes shared an 80% overlap in protein detections, of which quantitative measurements were strongly correlated (R^2^ = 0.8) [116]. Of course, any conclusions from this meta-analysis would require rigorous validation, but it does demonstrate that comparisons of independently-generated tissue proteomic datasets will be possible in the future [92]. Cross-referencing proteomic data findings with other public datasets such as The Cancer Genome Atlas (TCGA) and Human Protein Atlas [117] instills further confidence in biomarker identifications.

### Spatial resolution in discovery proteomics

One factor to consider in quantifying changes in the tissue proteome is tumour heterogeneity. This was recently shown by sampling the proteome of frozen prostatectomy specimens by SWATH-MS from benign prostatic hyperplasia and adenocarcinoma patients. To account for inter-patient, intra-tissue and inter-tissue variability, tissue biopsy sections were investigated from different areas of the same sample, different samples and different patients. It was noticed that proteins involved in DNA repair pathways differ significantly between different areas of the same tumor tissue, owing to tumour heterogeneity. Thus, it was suggested to account for this variability in future studies when identifying protein biomarkers from tissues [90]. Efforts are being made to distinguish changes in protein expression originating from disease progression from those originating from tissue heterogeneity and secondary biology pathways. To overcome this, the development of laser-capture microdissection (LCM) provides a powerful tool to isolate specific regions of a tumour cross-section. LCM was used to isolate neoplastic islands and stroma from the tumour front and inner tumour of oral cancer tissues. Discovery-based proteomics revealed the lower expression of cystatin-B in the islands which was further verified by IHC. The study was extended to targeted methods to further distinguish patients with increased chances of lymph node metastasis [118]. Increasing the throughput of LCM to match that of discovery-based proteomic experiments with increasing cohort size is imperative for this technology to transfer to the clinic.

Another MS-based technique that provides spatial information and shows potential in the clinic is Mass Spectrometry Imaging (MSI). MSI has already been established in the detection of metabolomics-based markers, as is making strides in the proteomics sphere. A sectioned tissue biopsy is surveyed by MALDI-MS at ever-increasing spatial resolutions, as low as a few micrometers [119], and routine analysis at 100 µm. As the laser moves over the tissue, a protein marker’s intensity is correlated to a colour-coded heat map. Clinical MSI experiments are most commonly reserved for the detection of small molecules [120], glycans [121] and lipids [122] due to caveats in proteomics studies related to spectral complexity, difficult characterization of distinguishing peaks, and poor ionization of in-tact proteins. More recently, peptide ionization can be facilitated by the brief digestion of surface proteins prior to MSI. One applicable area of interest that has caught the attention of cancer research is the ECM proteome, as it relates to tumour metastasis and treatment resistance. It has been shown that pre-digestion of liver and colon cancer sections with matrix-metalloproteinase prior to MSI gives further insights into how these enzymes modulate ECM [123]. Alternatively, MSI can determine the distribution of cancerous and healthy cell types in heterogeneous tissue cross-sections [124].

This tumour heterogeneity creates the need for single-cell resolution in proteomics. Fluorescence-activated cell sorting (FACS) is an antibody-based technique used to sort a heterogeneous mixture of cells based on the expression of predetermined protein markers but comes with low multiplexability of markers. An MS-based alternative has emerged, called mass cytometry, and allows for dozens of protein markers to be monitored in individual cells. This is accomplished by coupling antibody probes to unique stable heavy metal isotopes rather than fluorophores [125]. The cells are nebulized by inductively-coupled plasma (ICP) and the metal ions provide the mass spectrometer a quantitative readout of a marker’s distribution within a sample, such as immune cell infiltration [126]. For example, single-cell mass cytometry was used to quantify a panel of 73 proteins related to tumour-immune cell signalling in 144 breast tumours and 50 healthy tissue samples. Higher grade tumours were found that have exhausted T cell counts and higher frequencies of PDL1^+^ tumor-associated macrophages [127]. The mass cytometry quantitative strategy can also be extended to intact tissue sections like MSI. Imaging mass cytometry was used to visualise the tumor microenvironment in FFPE CRC tissues with a focus on immune cell infiltration [128] or more recently to evaluate the single-cell pathology landscape of a large cohort of 352 breast cancer tissues [129].

### Proteogenomics

Potentially the most powerful application of tissue proteomics to cancer research is using it in a concerted effort to complement genomics. This rapidly growing field is termed proteogenomics [130, 131]. Genomic research has significantly contributed to our understanding of cancer biology, through the identification of various cancer driver genes. It will be interesting to evaluate how some of these genomic aberrations modulate the cancer proteome. However, discovery proteomic analysis is dependent on reference databases of known peptides. Next-generation sequencing technologies can now provide comprehensive human genomes and transcriptomes that can detect single nucleotide polymorphisms and translocations. These variants are translated to unique proteoforms that would otherwise go undetected by traditional canonical sequence databases. Currently, MS-based detection of genomic aberrations is limited to only few cancer-specific variants, based on genomic estimations, due to the relatively low sequence coverage for each detected protein in a typical shotgun proteomic experiment. Future improvements in proteomics and computational approaches, such as open search algorithms like MSFragger [132], are expected to improve these detections. Alfaro et al. was able to combine publicly available databases with sample-specific genomic and transcriptomic data to interrogate proteomic data across 59 NCI cell lines. This resulted in the detection of 4771 mutations in 2200 gene products that would have otherwise escaped detection. This highlights the need for public availability of MS proteomics data so that expanded variant databases can be used for re-interrogation. In another example, Dimitrakopoulos et al. compared the DNA exome variants in 21 ER-positive breast cancer tissues to the proteomic data from the same samples [133]. This study demonstrated the limitations that still exist in the field as only 0.4% of these variants were detected at the proteome level. It was noteworthy that these detected variants belonged to the 6.3% most abundant mRNA transcripts which translated to many of the most abundant proteins. Nonetheless, the small subset of detectable variant peptides provides optimism that this class of potential biomarkers are well worth investigating in the future. Adding these variants to existing databases will make them more complete.

While the cancer genome and transcriptome of many cancers have been well elucidated, the cancer proteome and its relation to up-stream genomic alterations are poorly documented. In recent years, a growing number of studies have begun to integrate all levels of omics data to describe a comprehensive multi-omic assessment of tumours. These projects often require large collaborations between laboratories with unique skill sets, such as The Clinical Proteomic Tumor Analysis Consortium (CPTAC). This network was created by the National Cancer Institute to promote collaboration in an effort to accelerate our understanding of the molecular basis of cancer, with an initial focus on ovarian, breast and CRC (additional cancers have since been added). The consortium has produced increasingly complex multi-omic datasets, using standardized methods and applying them to growing cohort sizes of diverse surgical tissues. Stemming from the consortium, the Applied Proteogenomics Organizational Learning and Outcomes (APOLLO) network was created to bridge the CPTAC findings so that proteogenomic analysis becomes a routine component of personalized medicine. The International Cancer Proteogenome Consortium (ICPC) also works to bring cancer researchers together to share and compare data across 10 countries. The benefit of these networks is the validation of results in multiple centres. Irreproducibility in proteomics arises from differences in peptide digestion, pre-fractionation, chromatography, MS configuration and bioinformatics [134]. These aforementioned collaborations work to limit these factors with standard protocols and publicly available databases as detailed in the studies below, and summarized in Table 2.

**Table 2:** Summary of the clinical proteogenomic studies highlighted in this review

Protein quantitation	Tissue type	Additional omic datasets	MS model	Sample preparation	Clinical question	Proteins detected	Patient cohort	References
Label-free DDA	FFPE	GEN, TRA, PHO	OF	FASP, RPF	Stratify HBV-related hepatocarcinoma into subtypes	9252	110	[137]
	FF	EPI, TRA, PHO	OF	Urea, RPF, Super-SILAC	Distinguishing between four subgroups of medulloblastomas	3892	41	[140]
		GEN, EPI, TRA	QE	TFE	Biomarkers of curable PCa	7054	76	[6]
	OCT	TRA, EPI	OV	TFE	CRC characterization	7526	95	[4]
		GEN, TRA, PHO	QE	Urea, RPF, TMT additional	Tumour, adjacent healthy tissue and blood in colon cancer patients	8067	110	[79]
Label-free SWATH	OCT	TRA	5600 TOF	Pressure cycling technology, urea	Protein degradation rates in PCa and adjacent healthy tissue	3056	68	[91]
	FF	GEN, EPI, TRA	5600 TOF	RIPA buffer	Untreated and castration-resistant PCa compared to benign	4601	38	[138]
Isobaric Labelling	FFPE	GEN, TRA	QE	FASP and IEF, TMT	Recapitulating breast cancer subtypes	9995	45	[135]
	FF	GEN, TRA, PHO	QE HF	SDS, FASP, RPF, TMT	Tissue and blood samples from HBV-related hepatocarcinoma and healthy adjacent liver patients	10,783	159	[139]
		GEN, TRA	QE	FASP and IEF, TMT	Characterizing pathogenetic impact of hyperdiploidy in acute lymphoblastic leukemia	8480	89	[142]
		GEN, TRA, PHOS, GLYCO	QE	SDS, RPF, iTRAQ	Characterization of gastric cancer from tumour, healthy adjacent tissue and blood samples	9625	80	[141]
	OCT	GEN, TRA, PHO	OF Lumos	Urea, Basic RPF, TMT	Characterization of treatment-naïve clear cell renal cell carcinoma	11,355	103	[139]
		GEN, TRA, PHO	LTQ Velos	TFE. iTRAQ	High-grade serous ovarian carcinoma characterization	9600	174	[5]
		GEN, TRA, PHO	QE	Urea, basic RPF, iTRAQ	Characterization of breast cancer subtypes: basal, HER2-enriched, luminal A, luminal B	12,405	77	[3]

Proteogenomic studies on breast cancer have recently demonstrated the ability to narrow down candidates for driver genes and to identify therapeutic targets. Mertins et al. [3] quantified the proteome and phosphoproteome of 105 breast cancer tissues which had previously been genomically annotated in a TCGA study. Trans-omic connections were made between cancer-centric pathways including CETN3 leading to elevated EGFR levels, or SKP1 loss leading to increased SRC expression. In another study, Johansson quantified nearly 10,000 proteins across all 45 breast cancer tumours prepared by FASP. Encouragingly, breast cancer subtypes were recapitulated by the proteomic data and among the subtypes with poor prognosis, and further classification was possible based on proteins related to immune infiltration. This study also included the mapping of protein products to non-coding genes which opens the door to new, tumour-specific, immunotherapeutic targets [135].

Human hepatocellular carcinoma has been investigated by a couple of proteogenomic studies. In collaboration with CPTAC, Gao et al. [136] applied TMT 11-plex to assess the proteome expression profiles from 165 patients with hepatitis-B virus-related hepatocellular carcinoma. Pathways related to tumour microenvironment regulation, cell proliferation and metabolic reprogramming, were studied in greater detail, and PYCR2 and ADH1A1 were identified as prognostic indicators of further patient subtyping. This study provides a valuable resource moving forward as the field acts to better understand liver cancer biology [136]. A group from the Chinese Human Proteome Project (CNHPP) consortium used proteomic and phosphoproteomics to stratify a cohort of 110 paired hepatocellular carcinoma and non-tumour tissues into subtypes with different clinical outcomes [137].

Sinha et al. [6] investigated the proteogenomic landscape of PCa through quantitation of the genome, epigenome, transcriptome and proteome. Label-free analysis of 76 localized, intermediate risk prostate tumours led to the quantitation of 7000 protein groups. This study led to several interesting observations. First, that established genomic subtypes of PCa converge on five proteomic subtypes, which are themselves associated with clinical outcomes. That ETS fusion genes, the most common mutation in prostate tumors, perturb the proteome and transcriptome in dramatically divergent ways, particularly influencing metabolic pathways. Similar to studies from CPTAC, that RNA abundance explains only ~ 10% of variability in protein levels in PCa, but there is a broad network of trans effects that converge on specific functional pathways and unique to this paper that biomarkers comprising genomic and proteomic features significantly out-perform those comprised of either molecular feature alone. Latonen et al. [138] also performed integrative proteomics in fresh frozen PCa tissues using a SWATH-MS strategy and also reported aberrations in the proteome cannot be reliably predicted by other omics datasets including gene copy number, DNA methylation and RNA expression. Similarly, in a 110 clear cell renal cell carcinoma study, it was reported that a handful of genes demonstrated an expected decrease in protein expression when the gene was increasingly methylated such as IQSEC1. On the other hand, this was not the case at the transcriptomic-level (mRNA), suggesting post-translational regulatory mechanisms were at play [136, 139].

Other notable cancer proteogenomic studies include the study of medulloblastoma [140], early-onset gastric cancer [141], lymphoblastic leukemia [142], and ovarian cancer [5]. Additionally, CPTAC has released proteogenomic datasets for colon and rectal cancers [4, 79]. While understanding cancer biology is critical, other studies have focussed on observing proteogenomic aberrations that may affect anti-cancer treatment responses. For example, CRC patients often receive treatment with anti-EGFR monoclonal antibodies. Yet, a study mined transcriptomic and proteomic data to validate that wild-type KRAS is necessary in tumours for effective treatment due to variant peptides [143]. Moving forward, the integration of proteomic datasets with genomic-level data will become increasingly common in future research of oncology and personalised medicine.

## Proteomics of human body fluids

### Blood-based proteomics

The promise of liquid biopsies is that they are thought to provide proteomic information representative of a given tumour or tissue type but can be collected in a less-invasive and longitudinal manner. In clinical laboratory assays, blood is the most widely used human body fluid in disease diagnosis, prognosis and treatment outcomes. Blood consists of cellular components (i.e. erythrocytes, thrombocytes, and lymphocytes) and a liquid component called plasma [144]. Blood is tested for various plasma proteins via enzymatic assays or antibody-based immunoassays. Plasma has a wide dynamic range of more than ten orders of magnitude in protein abundances, with only 22 proteins constituting 99% of the protein content [145]. These protein concentrations range from serum albumin (50 mg/mL), immunoglobulins and coagulation factors down to small protein hormones and cytokines (pg/mL) [144]. This large dynamic range has made it difficult to study the plasma proteome due to the masking of often low abundant potential disease biomarkers by the few very highly abundant proteins. Advances in MS-based proteomic detection technology and sample preparation have helped to partially overcome these issues.

Immunodepletion and fractionation has expanded the number of detected plasma proteins into the thousands [146]. Immunodepletion of high abundance proteins can be achieved through immunoaffinity-based [147–149] or dye-based depletion [150, 151]. Depletion methods have many limitations including off-target capture of other proteins or the depletion of proteins bound to abundant proteins like albumin [151]. More recently, Geyer et al. [152] performed a single-shot label free proteomic strategy from 1 µL of blood plasma through a single finger prick, without immunodepletion that resulted in detection of over 300 proteins. These included more than 40 FDA-approved biomarkers, inflammatory markers and gender-related proteins with high reproducibility.

After establishing the human proteome atlas project (HUPO) in 2001 [153], the Human plasma proteome project (HPPP) was initiated in 2002. The collaboration of 32 labs across 13 countries aims to generate an open source data repository of the human plasma and serum proteome via MS [154]. Additionally, the project evaluates various sample preparation workflows, MS instrumentations and analysis platforms across different laboratories. The multi-centre data was integrated and resulted in 9000 proteins detected by one or more peptides, or 3020 proteins detected more stringently by two or more peptides [154]. HPPP provided the first initiative for characterizing the human plasma proteome. More recently, Geyer et al. [155] acquired deep proteomic data of whole blood, platelet-enriched plasma and erythrocytes from 20 individual samples and compared it with the established plasma and serum proteomes. This resulted in the detection of more than 6000 proteins, and insights into the proteome of each blood compartment. The aim of the project was achieved in establishing a reference proteome which could identify the proteins from contaminating cell types and plasma.

Various studies have investigated the blood (plasma and serum) proteome from patients with various types of cancer, as summarized in Table 3. In one of these studies, Pan et al. [156] analysed the blood from healthy control and pancreatic cancer samples. The serum and plasma were isolated, immunodepleted and labelled with light and heavy-labelled acrylamide. The samples were pooled, fractionated by reversed-phase LC, tryptic digested and further fractionated by strong-cation-exchange (SCX) chromatography. This detected 1300 different proteins with several proteins differentially expressed in cancer compared to controls. Several of these differentially expressed proteins were confirmed using ELISA in an independent sample cohort with strong correlation to MS quantitation [157]. Proteins like TIMP1, ICAM1, AZGP1, APOA2 and LTF showed better predictive power than CA19-9 in differentiating the pancreatic samples from healthy controls, which is a gold standard blood biomarker for pancreatic cancer.

**Table 3:** Summary of the urine and blood-associated clinical proteomic studies highlighted in this review

Liquid biopsy	Protein quantitation	MS model	Sample preparation	Clinical question	Proteins detected	Patient cohort	References
Plasma	Isotopic label	LTQ Orbitrap	Immunodepletion, filtration, SCX fractionation	Pancreatic cancer, pancreatitis and healthy control plasma	1300	3	[156]
	Label-free DDA	LTQ Orbitrap & 5500 Q-trap	PDX, N-glycopeptide enrichment, SRM validation in human sera	Ovarian cancer biomarker development	906	224	[175]
	Label-free SWATH	5600 TOF	Immunodepletion, SCX, SAX, RPF, size-exclusion chromatography	Early diagnosis of CRC	427	100	[159]
		5600 TOF	N-glycopeptide enrichment, Off-gel fractionation	Five different cancer types and their matched controls	1151	284	[172]
Serum	Label-free DDA	LTQ Orbitrap	PDX, N-glycopeptide enrichment, Targeted validation in human	PCa diagnosis	775	8	[173]
		QE HF	PDX, N-glycopeptide enrichment, PRM validation in human sera	High grade serious ovarian cancer biomarkers and longitudinal monitoring	2200	20	[87]
	iTRAQ	5600 TOF	Immunodepletion, SWATH verification	Proteins leaving lung cancer tumours into pulmonary veins	1000	50	[158]
Urine	Label-free DDA	5600 TOF	Gel fractionation, RPF, IEF	Characterization of the healthy urine proteome	6085	24	[176]
		QE	MW-filtration, SCX, PRM validation	Renal cell carcinoma prognostic biomarkers	2589	115	[183]
		LTQ	Gel fractionation	Identify novel therapeutic targets for Wilms tumour	6520	49	[184]
		QE	Gel fractionation	Profiling urine from lung cancer patients and other tumors	7408	46	[185]
Post-DRE urine	iTRAQ	OV	Ultracentrifugation, RPF	Discovery of new biomarker for high Gleason PCa	4710	18	[191]
	Label-free DDA	QE	Ultracentrifugation	Characterizing EVs from EPS in urine from PCa and healthy patients	877	24	[189]
	SRM	TSQ Vantage	MW filtration, TFE	Targeted proteomics identifies signatures for extracapsular prostate cancer	232	74	[188]
		Qtrap5500	FASP	Biomarker validation for early detection and stratification of PCa	64	107	[192]

In a squamous cell lung carcinoma study, blood samples coming from the pulmonary artery and vein of noncancerous and cancerous lung regions were obtained during surgery. The plasma was immunodepleted and fractionated and peptides were quantified by iTRAQ. The results showed 50 proteins to be abundant in the vein draining part of the cancerous regions compared to the noncancerous sectors [158]. In another study done by Ahn et al. [159] one hundred plasma samples from different stages of CRC and healthy controls were pooled to create a SWATH library. Similarly, the abundant proteins were depleted and peptides were further fractionated. In total 37 proteins were differentially expressed in higher stages of CRC, and seven of these proteins were further validated by ELISA and western blot analysis.

Circulating tumor cells (CTC) are tumor cells that shed from the primary tumor into the circulatory system and can lead to metastasis in different organs, but provide a unique source of potential biomarkers [160]. The occurrence of CTCs in blood is quite rare, which is estimated to be one CTC per millilitre [161]. Different approaches have been reported for the isolation and detection of epithelial based CTCs through antibody-based EpCAM-coated ferromagnetic beads. This method has been FDA-approved for advanced PCa [162], breast cancer [163] and CRC [164] metastasis studies. The other CTC isolation methods include chip-based isolation [165, 166] and MagSweeper [167]. Alternative methods like micro-fluidic immunofluorescence [168] and microfluidic western blotting [169] detect a limited number of proteins in the CTC samples. Advances in MS-based analysis, especially recent single cell proteomics approaches [170] could potentially provide proteome-wide insights of these CTCs to identify novel protein markers for their detection and insights into tumour heterogeneity, cancer progression and treatment outcomes. Isolating minute amounts of these cells is a challenging task and all the methods mentioned above come with limitations such as leukocyte crosslinking [161], which leads to contamination of the target CTC proteome. Recent advancements in moving toward single-cell proteomics will improve coverage of the CTC proteome in future studies. A recent study by Li et al. [171] spiked varying amounts of MCF-7 cells in blood to mimic CTC amounts and were isolated with anti-EpCAM microbeads primed with antibodies specific to MCF-7 surface proteins. These captured MCF-7 cells were further prepared for LC–MS where 1327 proteins were detected from 50 spiked cells and 2026 proteins from 100 cells.

The plasma glycoproteome from various carcinomas were studied by Sajic et al. [172]. In this study, blood samples from 284 subjects from four different types of carcinomas (CRC, lung, PCa and pancreatic) and their own control groups were compared by SWATH-MS. This identified 1151 plasma glycoproteins from 4347 N-glycopeptides. Among the various expression similarities and differences that were revealed, an increased THBS1 expression was found to be common between all carcinomas. Cima et al. [173] analysed the effect of PTEN inactivation on the *N*-glycoproteome during PCa progression. The N-glycopeptides from serum and prostate tissue of wild-type and KO (PTEN) mice models were enriched and analysed by LC–MS. This generated a total of 757 *N*-Glycoproteins. The comparison between WT vs KO sera and tissue shortlisted 49 biomarker candidates. These candidate proteins were selected for SRM-based targeted assay in 143 patient serum samples (disease and control). In the end, 33 proteins were quantified by SRM in 80–105 patient samples. An additional nine proteins were validated by ELISA, totalling 39 proteins as potential PCa biomarkers.

In a recent study by Sinha et al. [87], potential biomarker discovery for high-grade serous ovarian carcinoma (HGSC) recurrence was performed using PDX models. Briefly, HGSC recurrent tumor tissues were engrafted into immune-compromised mice. Serum from unengrafted animals served as controls. *N*-glycopeptides were enriched from serum and tumor tissues using hydrazide chemistry and analyzed by label-free proteomics. This resulted in 3675 *N*-glycopeptides corresponding to 2200 proteins containing the Asn-X-Ser/Thr *N*-glycosylation sequon. Following species-assignment and bioinformatic prioritization the authors systematically developed targeted proteomics assays and applied them to two longitudinal cohorts of HGSC serum samples. This study reports four putative biomarkers for the early detection of HGSC recurrence. The study reports on a novel strategy for the discovery of tumor-derived proteins using a combination of *N*-glycoproteomics and PDX models. A similar study was performed by Hüttenhain et al. [174]. Genetically engineered ovarian cancer mouse models and control mice samples were used for the selection of N-glycoproteomic biomarker candidates which were quantified by SRM in 124 patient sera with epithelial ovarian cancer and 110 healthy controls. A protein signature consisting of IGHG2, L1CAM, THBS, DSG2 and LGALS3BP outperformed CA125, a known marker, in the detection of ovarian cancer.

### Urine-based proteomics

Urine is another commonly sampled human body fluid because it is produced in large volumes and can be easily collected in a non-invasive manner. From a proteome perspective, urine is less complex than blood, with a narrower dynamic range, and is less prone to proteolytic degradation allowing for more stable storage over longer periods of time [175]. Improvements to LC–MS instrumentation have led to a series of studies reporting an increasing number of proteins that constitute the healthy human urine proteome, with Zhao et al. [176] reporting more than 6000 proteins. This has caught the attention of organizations seeking non-invasive biomarkers, including HUPO which has dedicated the Human Kidney and Urine Proteome Project (HKUPP) specifically to the analysis of urinary biomarkers [177]. The challenges associated with urinary proteomics studies include inter-patient variability since urine protein concentrations depend on kidney filtration and reabsorption performance which greatly fluctuates within a population. Secondly, intra-patient variability needs further characterization because urinary protein concentrations are affected by time of day, exercise, diet and age. For example, a study used capillary electrophoresis (CE-MS) to profile the urinary peptidomes and found that the expression of 112 urinary peptides strongly correlated with age in both healthy and diseased groups (mostly originating from collagen, uromodulin and fibrinogen) [178]. As such, the planning and selection of patient cohorts is an important component of urine proteomics studies in the future. Lastly, targeted approaches are highly applicable to liquid biopsy proteomic studies but do come with some caveats. Fu et al. [179] highlighted that target selection is crucial for accurate quantitation when they quantified twelve uromodulin (the most abundant protein in healthy urine) peptides by SRM in urine samples. However, only four were robustly correlated with ELISA protein concentrations due to the unpredictable confounding factors of clinical samples such as proteoform complexity. The urine-based proteomic studies detailed below are also summarized in Table 3.

It is estimated that 70% of urine proteins originate from the kidney and urinary tracts. As such, this proximity makes urine a valuable resource for monitoring urinary tract cancers [180]. Currently, bladder cancer relies on the detection of urinary NMP22 [181] by ELISA but this test suffers from low sensitivity. A second ELISA test for BTA [182] lacks specificity since the protein is also detected at high concentrations in blood that would yield a false-positive result in patients with poor filtration performance. CE-MS was used by Frantzi et al. [45] to develop a panel of peptide markers that distinguish primary from recurrent urothelial bladder cancer. The multi-centre discovery cohort was followed by a validation cohort combining 1357 patients. Intensities were normalized by 29 internal standard peptides. The predictive power of the current standard, cytology data, was augmented when combined with this peptide panel. Ortiz et al. [183] applied a shotgun approach to the study of urines from 115 kidney cancer patients. While distinctions could be made between healthy and cancer patient samples, the analysis demonstrated further diagnostic power by revealing EHD4 expression is elevated in clear-cell renal cell carcinoma relative to a benign oncocytoma. These techniques have also been applied to childhood cancers. Wilms tumour is the most common form of childhood kidney cancer and gel electrophoresis was used to fractionate urinary proteins from 49 patients followed by LC–MS. After validation in a larger cohort by ELISA, it was determined that prohibitin can be used for early disease detection, non-invasive monitoring of disease progression and as a target to block chemoresistance [184].

The remaining 30% of urinary proteins are from the glomerular filtration of blood suggesting that urine can also provide insight into cancers of distant organs [180]. A multi-centre, multi-disease study analyzed the proteomes of 231 patients by label-free proteomics. Prior to tryptic digestion, the urine samples were denatured to further release proteins from the uromodulin network. From this study, a protein signature was developed that not only distinguished lung cancer patients from healthy controls and other benign lung conditions such as pneumonia, but it was also from the urinary proteomes of other cancer types including: lung, bladder, cervical, CRC, esophageal, and gastric cancers [185].

Since the urethra passes through the prostate, urine is a valuable source of PCa biomarkers. A desirable trait of PCa markers is that they originate from the prostate rather than from other organs in the male urogenital tract. The Early Detection Research Network (EDRN) has studied post-DRE (digital rectal exam) urines which has been shown to contain a trove of potential biomarkers indicative of PCa status, including a non-coding RNA transcript, PCA3 [186]. However, these proteins are often lower abundance in urine creating the need for targeted MS quantitation. This approach was used by Shi et al. [187] and demonstrated that it is feasible to accurately multiplex the quantitation of 10 low-abundance, PCa-associated proteins in clinical urines. While detection of these proteins is not as feasible for shotgun proteomics, urine can also be collected after a digital rectal exam (DRE), a standard diagnostic test for patients with suspected PCa (termed post-DRE urine). The idea is that a DRE expels a small amount of prostatic secretions, a fluid often referred to as expressed prostatic secretions [14] that can then be collected within urine. Post-DRE urine is usually collected as the first flow, first catch urine (~ 50 ml) following a DRE. Kim et al. [188] used a shotgun approach to identify 232 proteotypic peptides that were differentially expressed between organ-confined and extracapsular PCa in expressed prostatic secretions. These peptides were then used to systematically develop targeted proteomics assays for evaluation in post-DRE urines. Briefly, SRM-MS assays were developed using synthetic, stable-isotope labelled peptides and subsequently applied to two independent cohorts of post-DRE urines. Statistical approaches were applied to develop clinical predictive models for PCa diagnosis (PCa patients vs. controls) and prognosis (patients with organ-confined compared to men with extracapsular disease). This study provided evidence that computationally guided proteomics in combination with richly annotated urine cohorts can discover highly accurate non-invasive biomarkers [13, 188].

Urinary extracellular vesicles (EVs) have attracted significant interest in recent years [189]. Although many different types of EVs (i.e. exosomes, microvesicles, etc.) are naturally released from healthy cells, their rate of release and cargo expression are reprogrammed in cancer to promote proliferation and metastasis while modulating the tumour microenvironment and immune response. These nanovesicles can be collected from urine through differential centrifugation, sucrose gradient density ultracentrifugation or filtration techniques [190]. iTRAQ was used to quantify 3500 proteins from the EV’s in post-DRE urine of PCa patients and identified FABP5 as a potential high-risk PCa marker. This differential expression was further validated by MS in cell line models, MRM quantitation and IHC staining [191]. A similar study was performed by Sequeiros et al. [192] who used a targeted MS approach to quantify 64 EV proteins by SRM from a larger cohort of 107 post-DRE urines composed of healthy men and those suffering from low-risk and high-risk PCa. A combination of two proteins (ADSV and TGM4) distinguished PCa patients from healthy controls, while a panel of five proteins (CD63, GLPK5, SPHMPSA, and PAPP) accurately classified patients by risk group that would help guide further treatment. The diagnostic panel was further verified by tissue microarray in prostate tissues.

### Alternative liquid biopsies sources

Aside from blood and urine, there are a variety of alternative human body fluids that could potentially be used for biomarker discovery. Some of these non-conventional fluid samples are rich sources of organ-specific proteins due to their close proximity, but often come at a cost of invasive sample collection. Thus, they may not be as applicable to routine clinical practices such as early cancer detection or longitudinal monitoring of cancer progression. The aforementioned prostatic secretions (often referred to as expressed prostatic secretion—EPS) is a fluid naturally produced by the prostate. The EPS proteome is hence a rich source of prostate-derived proteins and this fluid’s proteome has been directly profiled in the absence of a urinary background [14, 193–195]. One limitation of EPS is that it is in general only collected prior to radical prostatectomy, making it not suitable for routine clinical assays. It nevertheless provides an opportunity to discover prostate-derived biomarkers that can be further evaluated in post-DRE urines by targeted proteomics assays. Similarly, cerebrospinal fluid (CSF) surrounds the brain and spinal cord providing mechanical and immunological protection but is not sampled as often as blood in brain cancer treatments due to its invasive collection. Nonetheless, CSF cannot be overlooked as a valuable biomarker source since it was recently demonstrated to contain more than 3300 total proteins, with an enrichment in brain-specific proteins [196]. Spreafico et al. [17] analyzed the CSF proteome of 40 patients and identified a panel of six proteins that distinguished metastatic pediatric brain cancer from healthy controls. Likewise, ascites fluid collection from the peritoneal cavity is invasive but it has been sampled to detect biomarkers indicative of malignant gastric cancer [197], ovarian cancer [18, 19], hepatocellular carcinoma [198], changes in the *N*-glycoproteome related to epithelial ovarian cancer [199], and the proteomes of tumour cells derived from post-chemotherapy ovarian cancers [200].

Meanwhile, there exists a subset of alternative bodily fluid types that can be collected more readily but their proteomes have not been characterized as thoroughly as more conventional body fluids. Tears provide an intriguing biomarker source and only recently have there been efforts to characterize its proteome [16]. Tears from both eyes of eight healthy controls were analyzed and unsurprisingly, a significant proportion of the identified proteins were enzymes [201]. Similarly to urine, a subset of tear protein expressions have been reported to correlate with age [202]. While tear proteins are sampled more commonly for ocular-related diseases, some efforts have been made to associate tear proteins with primary breast cancer using MALDI [203]. Multiple studies report characterization of the saliva proteome since it is a good proxy for oral cancers. These studies often focus on the most common oral cancer, oral squamous cell carcinoma, with more than 1000 proteins regularly detected in a sample of saliva [204]. Label-free quantitation identified 22 overexpressed proteins in oral cancer patient saliva with resistin correlating with advanced stage and metastasis [15]. Finally, proteomic studies of stool samples have shown that feces are another potential source of biomarkers. Most notably, CRC escapes early detection since existing IHC tests demonstrate limited sensitivity. Komor et al. [205] performed LC–MS/MS on a cohort of nearly 300 fecal samples from healthy controls, adenoma patients, and CRC patients. A panel of proteins included haptoglobin, LAMP1, SYNE2, LRG1, RBP4, FN1 and ANXA6 and was able to distinguish the cancerous patients from controls with a high degree of specificity and sensitivity. Haptoglobin was further verified as a biomarker in a large (n = 795) validation cohort by antibody-based assays. Similarly, Bosch et al. [206] used MS to detect 834 proteins in feces, of which 29 were statistically enriched in CRC patient samples. Combinations of these new potential biomarker candidates even outperformed the current IHC standard hemoglobin.

Moving forward, supplementary inter- and intra-patient variability studies will be needed to confirm proper sample collection protocols and patient selection. As clinical fluid sample preparation methods become more established and standardized, these alternative body fluid proteomes will be characterized in further detail as part of the search for robust non-invasive cancer biomarkers.

## Conclusions and future directions

In summary, recent advances in biobanking and proteomics technologies now enable the robust profiling of clinical samples to unprecedented depth. To become a mainstream technology in clinical laboratories, similar to well-established genomics assays, proteomics technologies and investigators must embark on large-scale, possibly multi-institutional validations studies. Targeted proteomics assays are likely to play a central role for the validation of tissue or fluid-based biomarkers. Technologies such as MRM-MS (or more recently PRM-MS) are already firmly established in clinical biochemistry laboratories around the world for the detection and quantification of small molecules. The establishment of protein assays is the next logical step, but requires rigorous assay development metrics and large richly annotated validation cohorts.

Recent proteogenomics studies have also demonstrated the complementarity of proteomics and genomics technologies. Interrogation of biomolecules along the central dogma are expected to provide novel biological insights, multi-omics biomarkers and possible novel drug targets. While a handful of impressive studies have been published in recent years these have mainly focused on technical aspects of proteogenomics. What is missing is proteogenomics studies with a clear clinical question and large tissue cohorts to arrive at statistically powered conclusions. Another significant bottleneck of proteogenomics is the current lack of appropriate analyses strategies. While the field is capable of generating these large datasets, computational analysis strategies will require further improvements. This will require close collaboration between genomics, proteomics and data science/statistics investigators.

A unique feature of proteomics technologies is the ability to detect subcellular localizations [207], protein complexes [208] and post-translational modifications [209]. These so-called proteoforms [8] must be directly detected at the protein level and cannot be simply predicted from upstream genomics/transcriptomics data. For example, proteogenomics technologies have recently demonstrated great utility in the area of immune-oncology, in particular for the detection of druggable tumor-specific antigens [210]. A major hurdle for the development of cancer vaccines and T cell-based immunotherapies is the direct detection of MHC-associated neoantigens. Proteomics technologies have been developed for their isolation and MS-based detection. As these technologies further mature, including the development of more advanced analysis pipelines, direct clinical impact is to be expected.

Moving forward, the field of clinical proteomics is likely to see a rapid expansion of clinical cohort sizes as a result of standardized, high-throughput sample preparation techniques. This will minimize the frequency of studies that suffer from statistical underpowering and improve efficiency of translating biomarker candidates and drug targets to clinical application. Proteomics will increasingly become a critical part of cancer systems biology that integrate multi-omics data from genomics, epigenomics, transcriptomics and PTMs. This will create demands for superior computing power to handle and analyze increasingly large amounts of data. Further improvements in MS instrument sensitivity and speed will make deep proteome coverage more regularly attainable, especially without the need for extensive pre-fractionation. Improvements in detection/quantitation levels will also allow clinical proteomics to expand towards minimal input material and single-cell proteomics. Finally, data analysis pipeline will continue to enable the detection of protein panels and signatures that provide more diagnostics and prognostic accuracy relative to singular markers. These advancements will all be required for MS-based clinical proteomics to reach its full potential in translating research discoveries to improvements to clinical practice.

## Data Availability

Not applicable.

## Abbreviations

ACN: Acetonitrile
ADC: Adenocarcinoma
APOLLO: Applied Proteogenomics Organizational Learning and Outcomes
CDX: Cell derived Xenografts
CE-MS: Capillary electrophoresis
CID: Collision-induced dissociation
CNHPP: Chinese Human Proteome Project
CPTAC: Clinical Proteomic Tumor Analysis Consortium
CSF: Cerebrospinal fluid
CRC: Colorectal cancer
CTC: Circulating tumor cell
DDA: Data dependent acquisition
DIA: Data independent acquisition
DRE: Digital rectal exam
ECM: Extracellular matrix
EDRN: Early Detection Research Network
ELISA: Enzyme-linked immunosorbent assay
EPI: Epigenetic
EPS: Expressed prostatic secretion
ESI: Electrospray ionization
ETD: Electron-transfer dissociation
EVs: Extracellular vesicles
FACS: Fluorescence assisted cell sorting
FAIMS: High-field asymmetric ion mobility spectrometry
FASP: Filter-aided sample preparation
FF: Fresh frozen
FFPE: Formalin-fixed, Paraffin-embedded
GEN: Genomic
GLY: Glycoproteomics
HCD: Higher-energy collisional dissociation
HGSC: High-grade serous ovarian carcinoma
HKUPP: Human Kidney and Urine Proteome Project
HPPP: Human plasma proteome project
HRAM: High-resolution, accurate-mass
HUPO: Human Proteome Atlas Project
ICPC: International Cancer Proteogenome Consortium
IEF: Isoelectric focussing
IHC: Immunohistochemistry
iRT: Indexed retention time
iTRAQ: Isobaric tag for relative and absolute quantitation
LC: Liquid chromatography
LCM: Laser capture-microdissection
LLOQ: Lowest limit of quantification
LOD: Limit of detection
mAb: Monoclonal antibody
MALDI: Matrix-assisted laser desorption ionization
MRM: Multiple reaction monitoring
MS: Mass spectrometry
MSI: Mass spectrometry imaging
MW: Molecular weight
OCT: Optimal cutting temperature
OF: Orbitrap Fusion
OV: Orbitrap Velos
PDX: Patient-derived xenografts
PHO: Phosphoproteomic
PRM: Parallel reaction monitoring
PSA: Prostate-specific antigen
PCa: Prostate cancer
PTMs: Post-translational modifications
PVDF: Polyvinylidene difluoride
QE: Q-Exactive
RPF: Reversed-phase fractionation
SAX: Strong-anion-exchange
SCX: Strong-cation-exchange
SDC: Sodium deoxycholate
SDS: Sodium dodecyl sulfate
SILAC: Stable isotope labeling with amino acids in cell culture
SPS: Synchronous precursor selection
sqCC: Squamous cell carcinoma
S-trap: Suspension trapping
SWATH-MS: Sequential Window Acquisition of All Theoretical Mass Spectra
TCGA: The Cancer Genome Atlas
TFE: 2,2,2-Trifluoroethanol
TIMS: Trapped ion mobility spectrometry
TMT: Tandem mass tag
TNBC: Triple negative breast cancer
TOF: Time-of-flight
TRA: Transcriptomic
